# Choice of Cell Source in Cell-Based Therapies for Retinal Damage due to Age-Related Macular Degeneration: A Review

**DOI:** 10.1155/2013/465169

**Published:** 2013-04-22

**Authors:** Sudhakar John, Sundaram Natarajan, Periyasamy Parikumar, Mahesh Shanmugam P, Rajappa Senthilkumar, David William Green, Samuel J. K. Abraham

**Affiliations:** ^1^The Mary-Yoshio Translational Hexagon (MYTH), Nichi-In Centre for Regenerative Medicine (NCRM), P.O. Box 1262, Nungambakkam, Chennai 600034, India; ^2^Aditya Jyot Eye Hospital, Plot No. 153, Road No. 9, Major Parmeshwaran Road, Opp S.I.W.S. College Gate No. 3, Wadala, Mumbai 400 031, India; ^3^The Light Eye Hospital, No. 39 D, By Pass Road, Dharmapuri Ho, Dharmapuri, Tamil Nadu 636701, India; ^4^Sankara Eye Hospital, Varthur Main Road, Marthahalli, Kundalahalli Gate, Bangalore 560037, India; ^5^Department of Biotechnology, Acharya Nagarjuna University, Guntur, Andhra Pradesh 522510, India; ^6^Faculty of Dentistry, The University of Hong Kong, Pokfulam, Hong Kong; ^7^Department of Clinical Research, Faculty of Medicine, University of Yamanashi, 1110 Shimokato, Chuo 409-3898, Japan

## Abstract

*Background*. Age-related macular degeneration (AMD) is a complex disorder that affects primarily the macula involving the retinal pigment epithelium (RPE) but also to a certain extent the photoreceptor layer and the retinal neurons. Cell transplantation is a promising option for AMD and clinical trials are underway using different cell types. *Methods*. We hypothesize that instead of focusing on a particular cell source for concurrent regeneration of all the retinal layers and also to prevent exhaustive research on an array of cell sources for regeneration of each layer, the choice should depend on, precisely, which layer is damaged. *Results*. Thus, for a damage limited to the retinal pigment epithelial (RPE) layer, the choice we suggest would be RPE cells. When the damage extends to rods and cones, the choice would be bone marrow stem cells and when retinal neurons are involved, relatively immature stem cell populations with an inherent capacity to yield neuronal lineage such as hematopoietic stem cells, embryonic stem cells, or induced pluripotent stem cells can be tried. *Conclusion*. This short review will prove to be a valuable guideline for those working on cell therapy for AMD to plan their future directions of research and therapy for this condition.

## 1. Introduction 

The retina is a complex multilayered structure composed of two components, a photosensitive layer made of rods and cones and the neural connections, and the other part being the retinal pigment epithelium (RPE) and its basal lamina called Bruch's membrane, which helps to maintain the integrity of the barrier between the choroid and the retina. Retinal degeneration occurs in different forms of retinal diseases including retinitis pigmentosa (RP), age-related macular degeneration (AMD), glaucoma, and diabetic retinopathy [1]. Age-related macular degeneration (AMD) is a complex disorder with multifactorial etiology affecting the macula of the eye and involves the retinal pigment epithelium (RPE), Bruch's membrane (BM), and choriocapillaris which results in progressive and irreversible loss of central vision [2]. Estimates from the World Health Organization (WHO) indicate that there are nearly 161 million visually impaired people worldwide, 37 million of which are blind, with a yearly increase of 1 to 2 million [3] and that the number of people with AMD will increase due to an increase in the ageing population [4]. Dry type of AMD or nonexudative AMD refers to the condition in which, due to advancing age, the retina accumulates waste material which leads to amorphous deposits termed as drusen and the retinal pigment epithelial cells degenerate leading to loss of central vision. Wet type of AMD or exudative AMD refers to the condition in which new blood vessels from the choroid grow into the subpigment epithelial and subretinal spaces due to loss of integrity of the Bruch's membrane and these new blood vessels are leaky, leading to edema, which progressively disrupts visual function [5]. In both types of AMD, loss of photoreceptors has been documented [6]. Studies have shown that there is diffuse loss of photoreceptors in nonexudative AMD and a severe loss of photoreceptors in exudative AMD, both of which dominated by the loss of rods. With regard to the retinal neurons, it has been reported that loss of ganglion cells is profound (nearly 47% of the ganglion cells are lost in end-stage disease) in exudative AMD while in nonexudative AMD, the ganglion cell layer is preserved. Though ganglion cells are preserved in nonexudative AMD, it has been indicated that their functioning may not be normal [7]. Thus nearly all the layers of the retina are affected by this condition.

From its first description in the medical literature in 1874, AMD continues to be a disorder with no permanent solution [8, 9]. Current therapeutic approaches to AMD include thermal laser photocoagulation, surgical approaches like excision or displacement, photodynamic therapy and antivascular endothelial growth factor (anti VEGF) therapies [10]. The search for an ideal therapeutic approach that would help restore permanent vision in AMD is continuing. In this regard, cell transplantation holds some of the greatest promise because it addresses the root cause of disease by replacing the dysfunctional cells with healthy ones. We will limit this review with regard to cell-based therapies for damage to the various retinal layers that occur mainly in the AMD.

## 2. Comparative Retinal Regeneration between Different Vertebrates

Development of regenerative strategies is usefully guided by studying the difference in the regenerative potential between organisms. Retinal regeneration takes place in a variety of vertebrates including fish, amphibians, birds, and mammals, although it is restricted to certain stages of development in most animals [11]. The urodelian amphibians have the ability to regenerate the whole retina even after complete removal of retina, the cellular source of regeneration being the RPE which transdifferentiates into retinal progenitor cells. The stem cell in the ciliary marginal zone contributes to the retinal regeneration in these animals. It has been shown that retinal regeneration occurs in the embryonic stages of anuran amphibians and avian embryos. Yoshii et al. [12] showed that *Xenopus laevis* retains the ability to regenerate the retina and lens following the surgical removal of theses tissues even in adult animals. In adult animal species such as fish, amphibians, and birds, partial retinal regeneration has been described. In addition to retinal stem cells or precursor cells in the ciliary marginal zone, the rod precursors or Müller glial cells are also a source of regenerating retina. In teleost fish, retinal regeneration occurs even after surgical removal of a portion of the central part of the retina [13]. Intrinsic retinal regeneration, which occurs in fish and chick embryos by the formation of new retinal neurons from the progenitor/stem cells residing at the ciliary marginal zone, does not occur in mammals including humans. In mammals, however, in response to retinal damage, müller glia will proliferate and give rise to neuronal cells but the regenerative capacity of müller glia is limited in mammals than that in fish and birds [14].

Hence, there arises the search for alternative cell sources to regenerate the retinal cells when there is a cell loss. There are different cell sources for retinal cell transplantation and we hypothesize that the choice of cell source should depend on the level of damage in the retina that occurs due to AMD. There are a range of cell sources ranging from adult retinal pigment epithelial cells, bone marrow stem cells (BMSCs), fetal stem cells, embryonic stem cells (ESCs), induced pluripotent stem cells (iPSCs), and so forth. In this short review, we herein describe the various cell sources, which can be considered for repair and regeneration of different grades of damage to the retina in AMD, for optimal regeneration and efficient utilization of cell sources.

## 3. Cell-Based Restoration of Damage to the Retinal Pigment Epithelium (RPE) (Grade I) Damage

**Figure 1:**
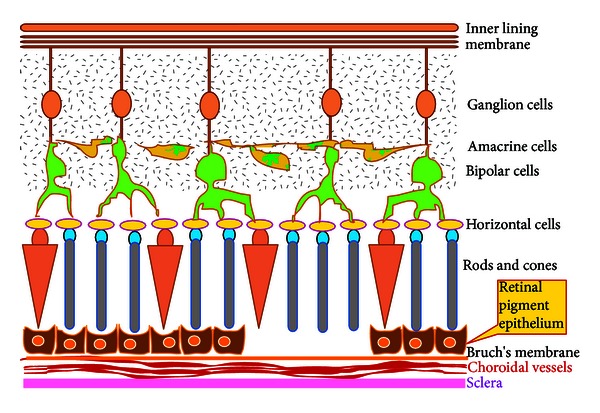
Pictorial representation of Grade I retinal damage. The retinal pigment epithelium (RPE) is degraded or absent. This can subsequently detrimentally affect the outer sensory part of the retina.

When the damage is restricted to the RPE layer (Figure 1), the choice we suggest would be RPE cells. In 1959, the first fetal retinal transplant into the anterior chamber of the eyes of rats was reported [15]. Cell culture experiments on RPE were carried out in 1980 [16]. Cultured human RPE cells were transplanted into the eyes of monkeys, first with open techniques and methods and later with closed cavity vitrectomy techniques [17–19]. The therapeutic potential of transplantation of the RPE was demonstrated at the Royal College of Surgeons in an animal model where a suspension of RPE cells labeled with carboxyfluorescein diacetate succinimidyl 5, 6-ester was injected in the subretinal space and the RPE cells were able to phagocytose the outer segments of photoreceptors [18]. In 1991, Peyman et al. [20] transplanted RPE in humans but the success rate was limited. Later, allogenic fetal RPE cell transplantation was tried in which immune rejection of the graft was a major problem. It has also been observed that the rejection rates were lower in dry AMD than that in wet AMD [21]. Autologous RPE transplantation is conventionally done employing two techniques, namely, RPE suspension and autologous full-thickness RPE-choroid transplantation [22–26]. Encouraging clinical outcomes has already been reported with the transplantation of the autologous RPE choroid from the periphery of the eye to a disease affected portion [25, 27, 28]. More than 30 homologous and 230 autologous RPE grafts have been performed till date [29]. A recent randomized controlled trial compared RPE sheet transplantation and RPE cell suspension injection. The trial concluded that outcomes with both approaches were comparable [30]. However, inability to transplant a uniform layer and formation of multilayered folds and contraction continue to be some of the challenges in RPE transplantation [17]. RPE cell suspensions also might not survive in an aged or defective host basal lamina after transplantation [31]. To overcome this difficulty, use of biologically derived basal lamina, amniotic membrane, Descemet's membrane, lens capsule and so forth have been suggested for transplanting the RPE as a sheet. The use of these biological scaffolds has the risk of biological contamination and disease transmission. Hence, polymers both natural and synthetic have also been tried for growing RPE cells in the form of a layer and transplanting as a RPE scaffold layer construct for better *in vivo* survival characteristics and improving the efficacy [31]. In this regard, an article by Lee et al. explores the microprinting of retinal pigment epithelial cells and iris pigment epithelial cells onto lens capsules and coating inhibitory molecules on the lens surface to control the organization of the cells growing on them [32]. In the case of autologous graft, the size of the full-thickness RPE graft that can be taken from the periphery of the same eye to patch the defect leads to insufficiency of the graft and for repeated RPE transplantation procedures, this approach is not feasible. Recently, the concept of retinal pigment epithelial cell expansion has been reported, in which a synthetic polymer scaffold has been used to support proliferation of the retinal pigment epithelial cells *in vitro*. This approach could offer a potential solution for the quantity of cells required for transplantation wherein a small quantity of RPE cells can be expanded for patient use and also in allogenic transplantation, RPE cells from a single cadaver retina can be expanded for use in many patients [33]. 

In a pilot study on 14 eyes (13 patients) with AMD who underwent subretinal surgery for treatment of foveal choroidal neovascularization, transplantation of retinal pigment epithelium harvested from the nasal subretinal area of the same eye was performed and a best-corrected visual acuity and satisfactory reading vision between Jaeger 1 and 4 were achieved in three eyes with no significant intraoperative or postoperative complications [34]. In a prospective trial on 56 patients who had foveal choroidal neovascularization (fCNV), subretinal surgery combined with simultaneous transplantation of autologous RPE cells resulted in improved visual acuity in eyes which received RPE transplantation compared to eyes which had membrane excision alone. These results provide evidence that transplantation of RPE may be regarded as a reasonable treatment option for AMD when the damage is limited to RPE layer.

However, the RPE transplantation is not without limitations. First, the extensive surgical intervention apart from requiring technical skills followed by a learning curve has its adverse outcomes as well. The technique such as neural retinal bleb detachment technique [35] has issues such as the surgeon's view of the foveal structures may be hampered and forcing the surgeon to work using one hand through the neural retinal hole which might expand during repeated manipulations. Moreover, the ability to separate the RPE layer alone without disturbing the choroid is another technical hurdle one has to keep in mind. The use of aged RPE cells whose function might not be optimal as that of young RPE cells is also of concern. Also autologous RPE in AMD may carry the same genetic information which might lead to recurrence of the disease [36].

To overcome the limitations of the neural retinal bleb detachment approach, the use of a 180 degree retinotomy to create a pedicled graft is recently being followed with good outcomes [37, 38]. This technique, since it allows the neural retina to be folded nasally offers a better visual operative field for the surgeon. The use of iPS cell technology to generate large numbers of RPE cells for transplantation [39] along with the feasibility to correct the genetic disease in the RPE cells created using iPS technology [40] offers exciting arenas for future research.

Use of RPE transplantation as a prophylactic procedure in AMD before neovascularization or geographic atrophy sets in can be considered as a step towards a permanent solution for AMD. This is advantageous because RPE transplantation has shown to stabilize the retinal vessels and prevents neovascularization [41]. This requires more refined surgical techniques, improved *in vivo* visualization, and research to develop RPE monolayers which will not be rejected after transplantation. As wet type of AMD is more difficult to treat with RPE transplantation compared to dry AMD, RPE cells themselves can be used as drug carriers for sustained release of anti-VEGF agents to prevent neovascularization in AMD. Nanoparticles have been employed to engineer the RPE cells to downregulate VEGF *in vitro* [42]. RPE transplantation can also be considered for patients who are unresponsive to anti-VEGF therapies. These are some areas of research which are worthwhile looking into, as we progress further in the field of RPE transplantation.

## 4. Cell-Based Restoration of Damage to the Retinal Pigment Epithelium and Rods and Cones (Grade II Damage)

**Figure 2:**
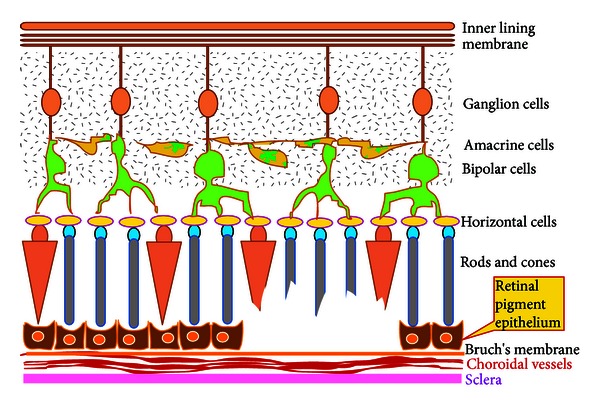
Pictorial representation of Grade II damage extending into the rods and cones.

If the damage extends up to the rods and cones (Figure 2), the choice of cell source would be BMSCs as there have been studies which have demonstrated the differentiation of BMSCs into photoreceptors [43–45] and also due to the fact that BMSCs are an established cell source for therapies. Studies on animal models have shown that the injection of BMSCs into the eye can potentially rescue injured retinal tissue [46]. It has also been observed that intravitreally injected adult bone-marrow-derived hematopoietic stem cells stabilize and rescue retinal blood vessels that would ordinarily completely degenerate in these “retinal degeneration animal models” [47]. In the retina, cellular differentiation in which the injected BMSCs might transdifferentiate to cells of the retina or fuse with the host cells, the bone-marrow-derived microglial cell formation and activation, which might clear away the cellular debris produced by retinal damage, production of neurotrophic factors thereby repairing retinal damage, are the proposed mechanisms of retinal repair by BMSCs. Paracrine effects like increasing angiogenesis, decreasing inflammation, activating neighboring resident stem cells, antiapoptotic and chemotactic signaling, and beneficial remodeling of the extracellular matrix are also implicated in the mechanism of how BMSCs contribute to retinal repair [48]. Clinical studies of intra-vitreal BMSC transplantation have been reported with the Jonas et al. [49] study in 2008 establishing safety of intra-vitreal injection of the autologous BMSCs in a 43-year-old patient with diabetic retinopathy. In 2010, the same group reported a study of autologous BMSC intra-vitreal injection in three patients with diabetic retinopathy, age-related macular degeneration, and optic atrophy [50]. However application of BMSCs for wet type of AMD is contraindicated, as the angiogenic potential of BMSCs would further worsen the condition. 

In addition to BMSC, the photoreceptor transplantation is another potential option for Grade II damage particularly in wet AMD where BMSC transplantation is not indicated. Pearson et al. demonstrated that the transplanted photoreceptor precursors in rod deficient mice were able to form the classic triad synaptic connections and visual signals could be generated by these transplanted rod photoreceptors [51]. Barber et al. were able to achieve integration of the transplanted photoreceptors across a range of inherited retinopathies [52]. These recent reports on the transplantation of photoreceptor cells for improving the vision may also be considered as a choice for damage extending to the photoreceptors, as more evidences gather. 

Though the above evidences are very much promising, when translating to human trials, one has to keep in mind the limitations of using BMSC derived photoreceptors or BMSCs per se or precursors of photoreceptors for Grade II damage to the retina, as the reproducibility of the parameters such as synapses formation, posttransplantation viability of the cells, and their integration with other layers of retina might vary between animal models which are young having undergone a controlled damage versus a human patient of a chronic pathophysiology.

## 5. Cell-Based Restoration of Damage to the Retinal Pigment Epithelium, Rods, Cones, and Retinal Neurons (Grade III Damage)

**Figure 3:**
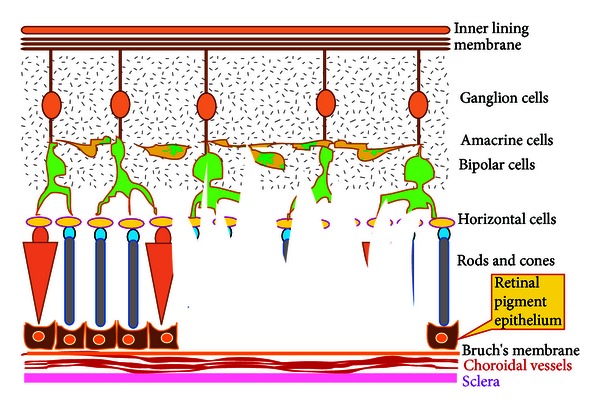
Pictorial representation of Grade III damage extending to the bipolar cells.

When the damage extends to the retinal neurons (Figure 3), a stem cell population, which has inherent capacity to give rise to neuronal lineage, is preferred. In this, we describe the various cell sources, which could give rise to the neurons of the retina. 

Developmentally, the nonneural retina and the neural retina share common origin from the optic vesicle. This characteristic makes RPE a viable source for generating retinal neurons. Experiments in chick embryos showed that the RPE cells in the eye, in explant, or in a dissociated cell culture can give rise to cells resembling retinal neurons when reprogrammed with appropriate regulatory genes involved in retinal neurogenesis. However, whether this can be applied to mammals can be answered only by future studies. Since the RPE is located adjacent to the neural retina, RPE reprogramming, if successful in mammals, may offer an approach to repopulate the neural retina [14]. Several studies on reprogramming retinal pigment epithelium to differentiate into retinal neurons with Sox2, Neurogenin 1, ash 1, and so forth have been reported with success [53–56]. Recently it has been reported that a subpopulation of adult human RPE cells can be activated *in vitro* to a self-renewing cell, the retinal pigment epithelial stem cell (RPESC) that loses RPE markers, proliferates extensively, and can redifferentiate into stable cobblestone RPE monolayers. These findings show the RPESC as an accessible source for retinal replacement therapy [57]. 

BMSCs, especially hematopoietic stem cells (HSCs), are another preferred source for generating neurons because mammalian BMSCs have been observed to differentiate into neural cells in several *in vitro* and *in vivo* studies [58–60]. It has been proven by studies that HSCs retains the capacity to differentiate into cell types like oligodendrocytes progenitors, ependymal cells, neurons, and astrocytes [61]. An animal study by Sigurjonsson et al. [62] proved that substantial proportions of adult human HSCs differentiate into full-fledged neurons in lesions of the developing spinal cord in the chicken embryo. In a laser-induced Bruch's membrane rupture mice model of choroidal neovascularization, it was demonstrated that reconstitution of GFP+ HSCs in lethally irradiated C57BL6/J mice resulted in GFP+ cells adopting the morphological and immunological characteristics of endothelial cells, pericytes, astrocytes, RPE, and macrophages, and the study also showed that these HSCs participate in repair of the CNV lesion [63]. In another study, intra-vitreal injection of mouse and human adult bone-marrow-derived lineage-negative hematopoietic stem cells resulted in neurotrophic rescue of retinal degeneration. Thus HSCs are attractive candidates for such Grade III damages to the retina [47]. 

Another choice would be human embryonic stem cells (hESCs), as two prospective clinical studies have started recently to establish the safety and tolerability of subretinal transplantation of hESC-derived RPE in patients with Stargardt's AMD and dry AMD. The finding of both studies suggests that the there was no sign of hyper proliferation, abnormal growth, or immune mediated transplant rejection in the patients. The best corrected visual acuity improved from hand motions to 20/800 in the study on the patient with Stargardt's macular dystrophy, and vision improvement was also observed in the patient with dry age-related macular degeneration [64]. Thus, human embryonic stem cells could also be considered for regeneration of the retinal layers when damage extends to the neurons. However, issues with hESCs like immune rejection, risk of teratogenicities, and ethical barriers need to be overcome and safety has to be firmly established before they are brought into routine clinical procedures.

There are several studies reported on generating retinal neural cells from induced pluripotent stem cells (iPSCs). Chen et al. demonstrated that iPSCs derived from mouse fibroblast inherently express the retinal progenitor cells-related genes and overexpression of Math5 with addition of Noggin can help in generation of retinal ganglion-like cells from these iPSCs [65]. In another study by Tucker et al., subretinal transplantation of retinal precursors derived from iPSCs resulted in these cells taking up residence in the retinal outer nuclear layer leading to increased electroretinal function [66]. Thus, iPSCs could also be a very good candidate for cell replacement therapy in AMD when the damage extends to the neural cell layers. The study by Zhao et al. [67], which reported that in contrast to derivatives of ESCs, some cells differentiated from iPSCs had an abnormal gene expression which can induce T-cell-dependent immune response in syngeneic recipients, and the studies, which showed that iPS cells show rapid telomere shortening, DNA chromosomal damage, and increased p21 expression that cause cell growth arrest, caution us from the use of iPSCs clinically but future research will witness the generation of “safe” as well as viable human iPS-derived somatic cells including RPE. 

It will also be worthwhile to consider the differentiation ability of neural stem/progenitor cells to retinal neurons which has been reported in studies. Therefore, if the damage extends to the retinal neurons, any cell population capable of differentiating into neuronal lineage can be considered, provided the capability of the transplanted cells either pluripotent stem cells like the hESCs, iPSCs, or neurons generated from HSCs or RPESCs in forming neuronal connections with the lateral geniculate ganglion or higher areas in the visual cortex is proven first by appropriate *in vitro* experiments followed by *in vivo* studies which though might be a long way ahead according to the authors but is worth considering.

## 6. Discussion

In this short review, the authors place their thoughts on the choice of cell source for transplantation to treat retinal damage depending on the extent of damage. This is important in the wake of multitude studies that have been reported and are being reported on cell-based therapies for AMD and other retinal diseases which creates a dilemma for the clinicians, and researchers to choose the right cell source.

While a question may arise as to how the extent of damage can be assessed, it should be understood that the level of damage might not be uniform throughout the retina even in the same patient and even within the same eye. The tissue damaged most can be deciphered based on the following.

1. Pathogenesis of the disease, the fact that AMD affects the macula and the RPE primarily, should be kept in mind and early disease versus late disease can help the clinician to hypothesize whether the damage would have involved the other layers too enabling further assessment.
2. Investigations that may aid to decide on the extent of damage are as follows.
  - Optical coherence tomography (OCTs) can give a picture of gross morphology of the macula. It gives an almost histopathological section of the retina and the area of tissue damage can be visualized using OCT but the functional damage cannot be assessed from this. Newer OCTs are capable of providing high tissue definitions.
  - Auto fluorescence, red-free imaging, fluorescein, and indocyanine angiograms can also give information about the extent of damage to the RPE, the outer and inner blood retinal barrier, and a clue about accumulation of fluid within the retina and subretinal space which can be indirect indicators of tissue damage.
  - Functional assessment is possible to a reasonable extent in the early stages of the disease with electrodiagnostic tests such as the ERG, multifocal ERG, and the electrooculogram.

The caveats are that damage to one tissue does result in damage to the other. For instance, damage to RPE will cause retinal degeneration over time and damage to the photoreceptors in turn can cause ganglion cell loss. Hence, it would be difficult to quantify the degree of damage to each of the tissue layers of the retina with certainty. However, importance is to be attached to the macula while assessing the extent of damage. Thus the etiopathogenesis of the disease, the severity, and clinical evaluation will be able to provide an idea of the damage to the retinal layers. 

Once the level of the damage has been assessed, the cell source most appropriate for regeneration of that particular layer must be chosen. The reason why RPE transplantation is suggested over other cell sources in case of damage mostly limited to RPE (Grade I damage) is because of the fact that a cell source for transplantation is available from the same tissue of origin. It is logical to conceive that use of cell source from same the tissue of origin, that is, RPE itself, is safe and is expected to provide better outcomes compared to cells from distant tissue or origin. Also RPE cells are a relatively mature population compared to other cell sources like bone marrow, embryonic stem cells, and so forth, which increase the safety and autologous transplantation of RPE from the same eye is also possible. 

Choice of BMSCs for damage extending to the photoreceptors (Grade II damage) would be based on the advantage of easy availability and accessibility in obtaining bone marrow and the fact that BMSCs have been established as a promising source of stem cells in regenerative therapies for a variety of diseases and disorders including cardiac diseases, liver cirrhosis, neurodegenerative disease, and bone and cartilage diseases [68–72]. Transplantation of photoreceptors or photoreceptors from human embryonic stem cells or induced pluripotent stem cells presents a possibility but the difficulties associated with the isolation and culture of these cell types along with the ethical issues surrounding the use of embryonic stem cells should be considered.

In the case of damage extending to the neurons, we have to consider using embryonic stem cells, HSCs, iPSCs, or cells with neuronal lineage differentiation capability as neuronal differentiation presents a complex phenomenon compared to retinal pigment epithelial or photoreceptor differentiation. 

This differential choice of cells for each grade of damage would help clinicians make the right choice in the appropriate situations as otherwise they may end up choosing inaccessible cell sources and cell sources with complex culture methodologies thereby leading to a waste of resources, time, and personnel involved in establishing that particular cell source for clinical application. Though, at this point, RPE transplantation for Grade I damage to the retina according to the authors is very much promising, the specific cell sources for Grade II and III damages need extensive research, while keeping in mind that the varying grades of damage in the clinical settings of a human patient might not be reproducible in *in vitro* models or animal experiments.

## 7. Conclusion

Thus, this paper provides valuable suggestions for researchers and clinicians on the appropriate cell sources for different grades of damages of the retina because identifying the right source would be the first step for a truly successful cell therapy. We understand that though several cell sources have been described in this paper, only some of the cell sources have entered clinical trials. Also among all approaches, the RPE transplantation approach has provided encouraging results and all other cell sources for repair, rejuvenation, or restoration of the degenerated retina need significant validation before they can be considered for clinical translation, which is worth an effort considering the light at the end of the tunnel.
